# Supplementary material for: A DVD-MoS2/Ag2S/Ag Nanocomposite Thiol-Conjugated with Porphyrins for an Enhanced Light-Mediated Hydrogen Evolution Reaction
Source: Nanomaterials (Basel). 2020 Jun 29;10(7):1266. doi: 10.3390/nano10071266 (PMC7408523; doi:10.3390/nano10071266)
Supplement: Supplementary file 1 [file nanomaterials-10-01266-s001.pdf]

Supporting Information

A DVD-MoS_2/Ag_2S/Ag nanocomposite thiol-conjugated with porphyrins for an enhanced light-mediated hydrogen evolution reaction.

Leonardo Girardi [1], Matías Blanco*[1,2], Stefano Agnoli[1], Gian Andrea Rizzi*[1] and Gaetano Granozzi [1]

[1] Department of Chemical Sciences and INSTM Unit, University of Padova, Via F. Marzolo 1, 35131, Padova, Italy. gianandrea.rizzi@unipd.it

[2] Department of Organic Chemistry, Universidad Autónoma de Madrid, Ciudad Universitaria de Cantoblanco, Calle Francisco Tomás y Valiente, 7, 28049 Madrid, Spain. matias.blanco@uam.es

* Correspondence: matias.blanco@uam.es; Tel.: +34 914975022 (M.B.)
* Correspondence: gianandrea.rizzi@unipd.it; Tel.: +39 0498275722 (G.R.)

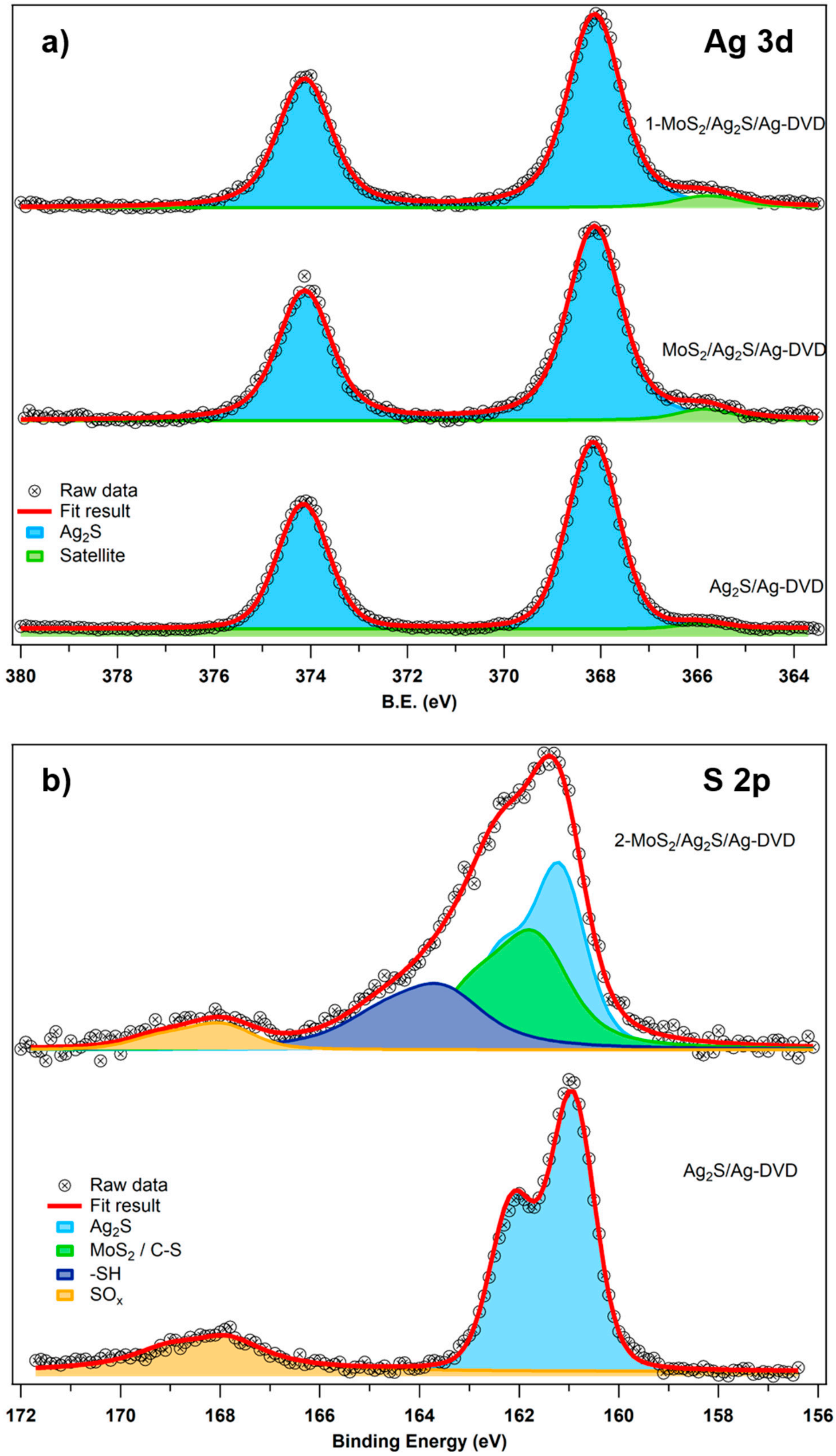

Figure S1. High resolution XPS spectra in the core level region of a) Ag 3d (samples Ag_2S/Ag-DVD, MoS_2/Ag_2S/Ag-DVD and **1-MoS_2/Ag_2S/Ag-DVD**) and b) S 2p, for the samples **2-MoS_2/Ag_2S/Ag-DVD** and Ag_2S/Ag-DVD .

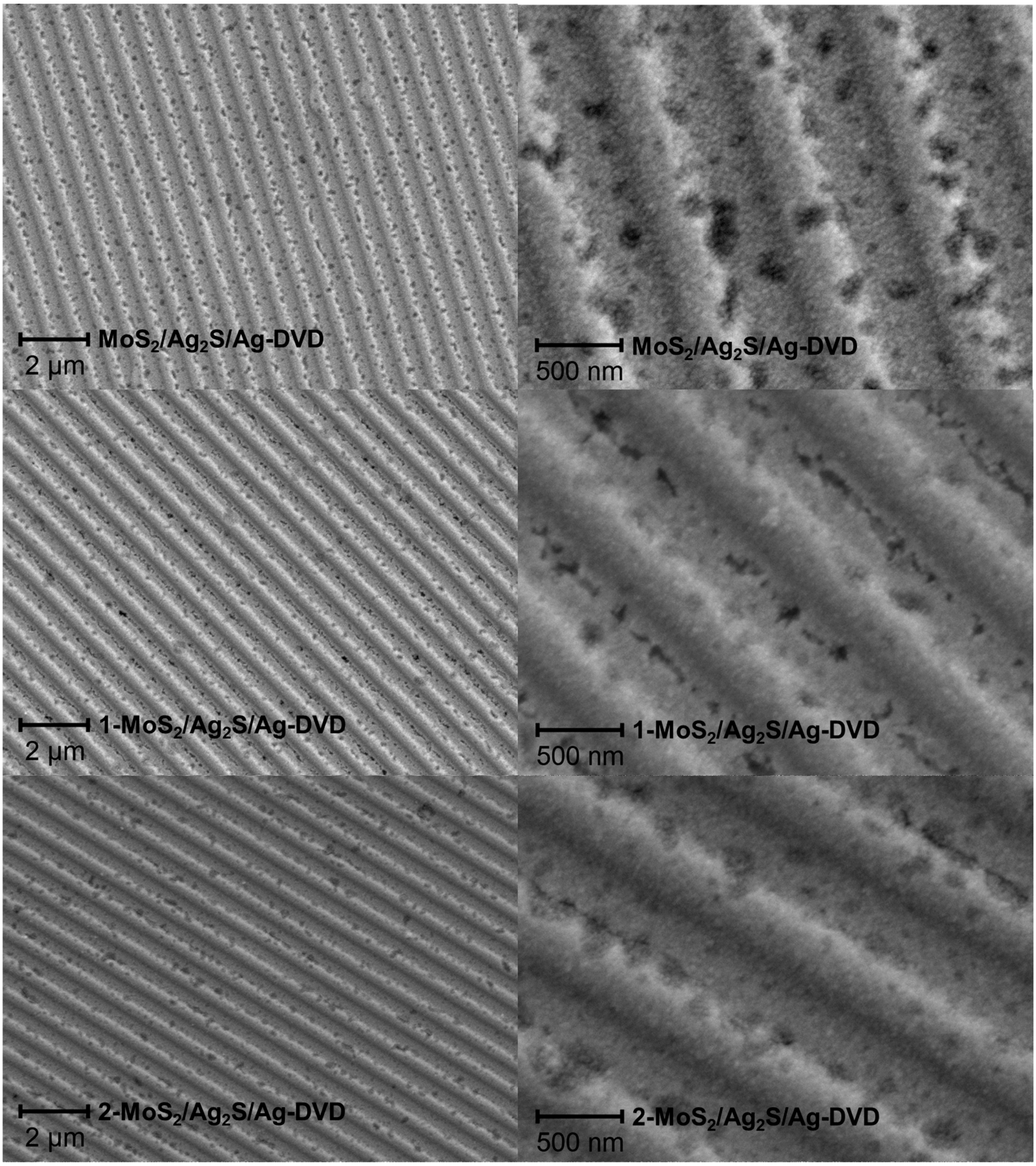

Figure S2. SEM images of the analysed samples.

It is clear that the porphyrin modification does not change the morphology of the material.

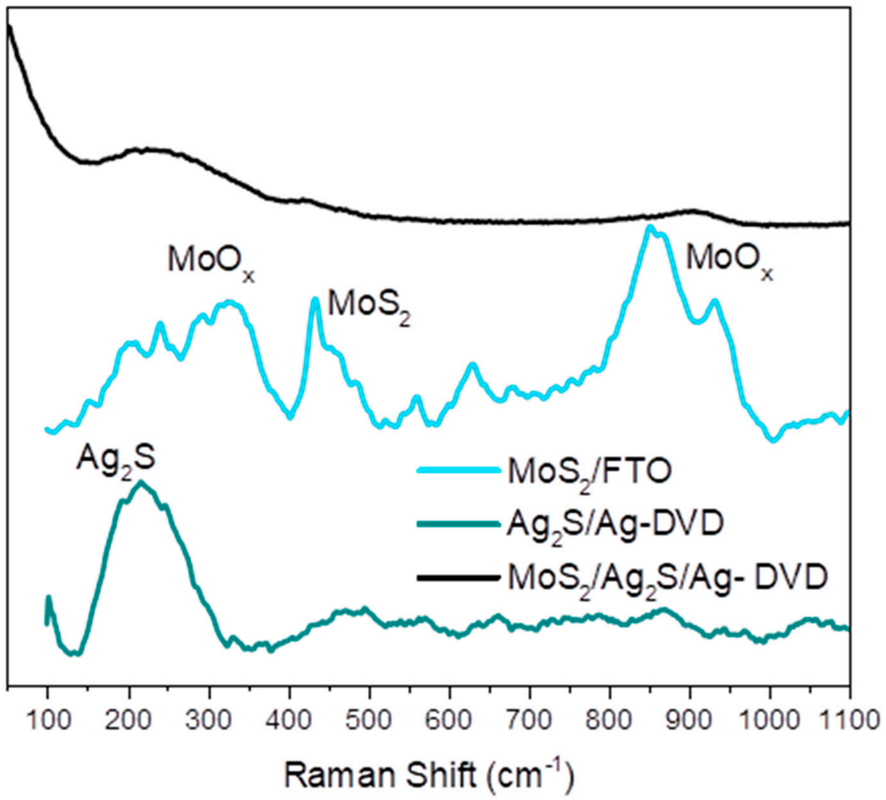

Figure S3. Raman spectra of MoS_2/Ag_2S/Ag-DVD compared with the sample MoS_2/FTO and Ag_2S/Ag-DVD.

It is clear that the characteristic bands of the two materials (Ag_2S 200 cm^{-1} and MoS_2 450 cm^{-1}) are present in the control MoS_2/FTO and Ag_2S/Ag-DVD spectra.

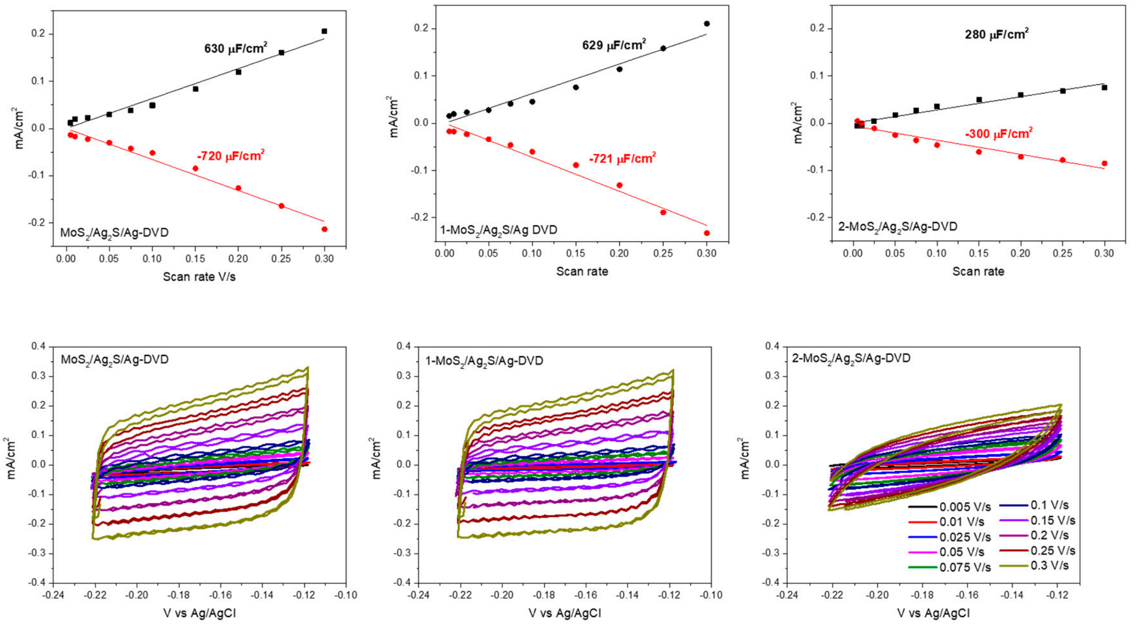

Figure S4. Cyclovoltammetry analysis in the capacity region for the samples as prepared and modified by the porphyrin 1 and 2.

It is evident that the samples coated with the molecule **2** present a higher hydrophobicity behaviour compared to the sample functionalized with porphyrin 1.

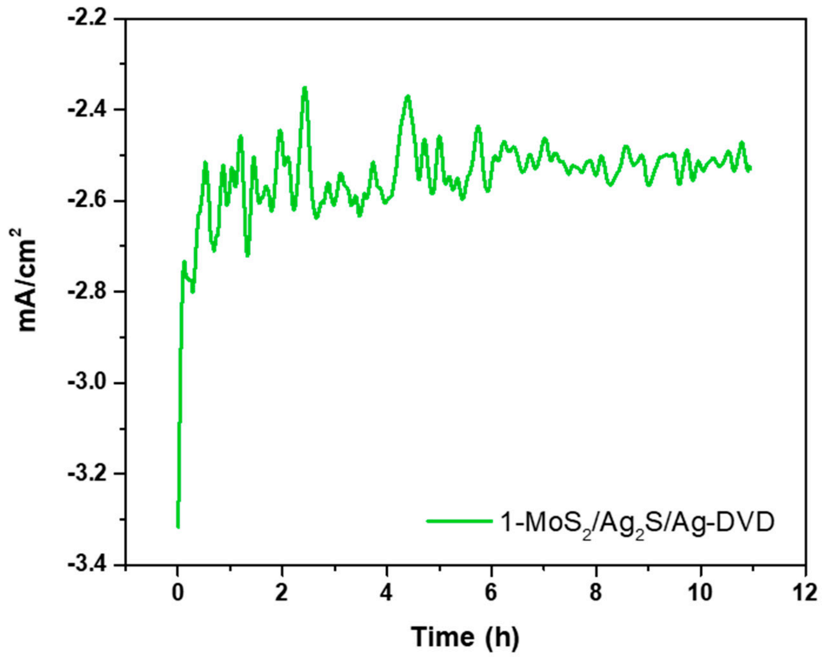

Figure S5. Chronoamperometry under constant illumination at -0.2 V vs RHE for 11 h.

Table S1. Comparison of HER performance of our hybrid material with state-of-the-art electrocatalysts

Catalyst	η_{10} [a]	Tafel Slope [b]	Ref.
1-MoS_2/Ag_2S/Ag-DVD	264	48.3	This work
Fe@CNT	77	40	S1
Cu-MOF - GO	87	84	S2
MoS_2 nanosheets	450	307	S3
Ni Foam	260	99.1	S3
g-C_3N_4 @ NG	240	51.5	S4
Pt @ DNA	26	30	S5
ce-$MoSe_2$	243	59	S6
MoS_2-PANI	200	45	S7
MoS_2-Polysaccharides	130	60	S8
Oxidized MoS_2	180	58	S9
Others	0 - 300	30 - 110	S10
a) Overpotential to reach a current of – 10 mA cm^{-2}; b) mV dec^{-1}			

References

S1. Mohammad Tavakkoli, Tanja Kallio, Olivier Reynaud, Albert G. Nasibulin, Christoffer Johans, Jani Sainio, Hua Jiang, Esko I. Kauppinen, and Kari Laasonen. Single-Shell Carbon-Encapsulated Iron Nanoparticles: Synthesis and High Electrocatalytic Activity for Hydrogen Evolution Reaction. *Angew. Chem. Int. Ed.* **2015**, *54*, 4535–4538. doi: 10.1002/anie.201411450.

S2. Maryam Jahan, Zhaolin Liu, Kian Ping Loh. A Graphene Oxide and Copper-Centered Metal Organic Framework Composite as a Tri-Functional Catalyst for HER, OER, and ORR. *Adv. Funct. Mater.* **2013**, *23*, 5363-5372. doi: 10.1002/adfm.201300510

S3. Jian Zhang, Tao Wang, Darius Pohl, Bernd Rellinghaus, Renhao Dong, Shaohua Liu, Xiaodong Zhuang, Xinliang Feng. Interface Engineering of MoS2/Ni3S2 Heterostructures for Highly Enhanced Electrochemical Overall-Water-Splitting Activity. *Angew. Chem. Int. Ed.* **2016**, *55*, 6702-6707. doi: 10.1002/anie.201602237.

S4. Yao Zheng, Yan Jiao, Yihan Zhu, Lu Hua Li, Yu Han, Ying Chen, Aijun Du, Mietek Jaroniec, Shi Zhang Qiao. Hydrogen evolution by a metal-free electrocatalyst. *Nat. Commun.* **2014**, *5*, 3783. doi: 10.1038/ncomms4783

S5. Sengeni Anantharaj, Pitchiah E. Karthik, Balasubramanian Subramanian, Subrata Kundu. Pt Nanoparticle Anchored Molecular Self-Assemblies of DNA: An Extremely Stable and Efficient HER Electrocatalyst with Ultralow Pt Content. *ACS Catal.* **2016**, *6*, 4660–4672. doi: 10.1021/acscatal.6b00965.

S6. Blanco, M.; Lunardon, M.; Bortoli, M.; Mosconi, D.; Girardi, L.; Orian, L.; Agnoli, S.; Granozzi, G. Tuning on and off chemical- and photo-activity of exfoliated $MoSe_2$ nanosheets through morphologically selective "soft" covalent functionalization with porphyrins. *J. Mater. Chem. A* **2020**, *8*, 11019 – 11030 10.1039/D0TA03302B, doi:10.1039/D0TA03302B.

S7. Nan Zhang, Weiguang Ma, Tongshun Wu, Haoyu Wang, Dongxue Han, Li Niu. Edge-rich MoS_2 Naonosheets Rooting into Polyaniline Nanofibers as Effective Catalyst for Electrochemical Hydrogen Evolution. *Electrochimica Acta*, **2015**, *180*, 155-163. doi: 10.1016/j.electacta.2015.08.108

S8. Ning Liu, Lichun Yang, Sinong Wang, Zhiwei Zhong, Sina He, Xiaoyun Yang, Qingsheng Gao, Yi Tang. Ultrathin MoS_2 nanosheets growing within an in-situ-formed template as efficient electrocatalysts for hydrogen evolution. *J. Power Sources*, **2015**, *275*, 588-594.

S9. Jinxue Guo, Fenfen Li, Yanfang Sun, Xiao Zhang, Lin Tang. Oxygen-incorporated MoS_2 ultrathin nanosheets grown on graphene for efficient electrochemical hydrogen evolution. *J. Power Sources,* **2015**, *291*, 195-200.

S10. Ali Eftekari. Electrocatalysts for hydrogen evolution reaction. *Int. J. Hydrog. Energy*. **2017**, *42,* 11053 – 11077. doi: /10.1016/j.ijhydene.2017.02.125
